# 180. Impact of a two-step diagnostic bundle on hospital-onset *Clostridioides difficile* infection rates and treatment across a large health system.

**DOI:** 10.1093/ofid/ofac492.258

**Published:** 2022-12-15

**Authors:** Radhika S Polisetty, Brian M Hoff, William J Moore, Michael Postelnick, Sheila K Wang, Teresa Zembower, Qi Chao, Michael Malczynski, Grace Barajas, Christina Silkaitis, Asra Salim, Jaime Borkowski, Rishita Shah, Andrea Quinn, Kyle Johnicker, Charlene Liang, Stephanie Chang, Sarah H Sutton

**Affiliations:** Midwestern University College of Pharmacy/ Northwestern Medicine Central DuPage Hospital, Winfield, Illinois; Loyola University Medical Center, Maywood, Illinois; Northwestern Medicine, Chicago, Illinois; Northwestern Medicine, Chicago, Illinois; Midwestern University College of Pharmacy/Northwestern Memorial Hosptial, Downers Grove, Illinois; Northwestern University, Chicago, Illinois; Northwestern Memorial Hospital, Northwestern University Feinberg School of Medicine, Chicago, Illinois; Northwestern Memorial Hospital, Northwestern University Feinberg School of Medicine, Chicago, Illinois; Northwestern Memorial Hospital, CHicago, Illinois; Northwestern Medicine, Chicago, Illinois; Northwestern Memorial Healthcare, Chicago, Illinois; NM Delnor Hospital, Geneva, Illinois; Northwestern Lake Forest Hospital, Lake Forest, IL, Illinois; Northwestern Medicine Palos Hospital, Palos Heights, Illinois; Northwestern Medicine Kishwaukee Hospital, DeKalb, Illinois; Marianjoy Rehab Hospital, Wheaton, Illinois; Northwestern Medicine - Huntley Hospital, Huntley, Illinois; Northwestern Memorial Hospital, CHicago, Illinois

## Abstract

**Background:**

Distinguishing true *Clostridioides difficile* Infection (CDI) from colonization is a challenge, with nearly 20% of hospitalized adults being carriers of *C. difficile*. Polymerase chain reaction (PCR) testing alone is not able to distinguish colonization from infection, leading to over-diagnosis and unnecessary treatment. Despite interventions including pre-approval by antimicrobial stewardship programs (ASP), reportable hospital-onset CDI (HO-CDI) rates across our health system remained high. In 2021, we implemented a *C. difficile* PCR with reflex toxin enzyme immunoassay (EIA) testing strategy to improve diagnostic accuracy and treatment outcomes. The purpose of this study was to evaluate the impact of this two-step testing algorithm bundled with education, ASP support and order set changes on HO-CDI rates and *C. difficile* treatment across our health system.

**Methods:**

PCR with EIA testing algorithm was implemented between May and August 2021 across seven hospitals within the Northwestern Medicine Health System. Multifaceted education was delivered to leadership and clinicians in person and electronically to. ASP performed daily diagnostic prospective audit and management support. Clinical decision support (CDS) was incorporated into order sets to promote diagnostic stewardship (Table 1, Figure 1). Standardization of analyst-developed tracking reports allowed for longitudinal monitoring across the system and at each facility, including unit- and patient-level data.

**Figure 1:**
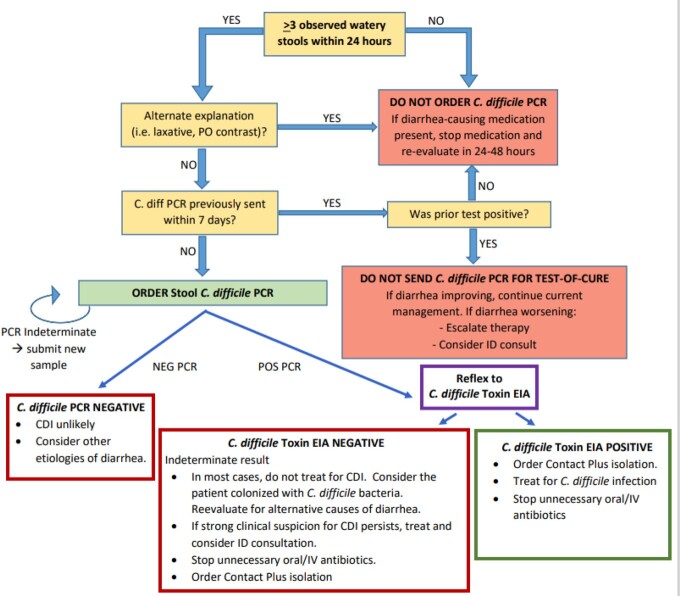
C. Difficile Testing Algorithm PCR/Reflex Toxin EIA

**Figure:**
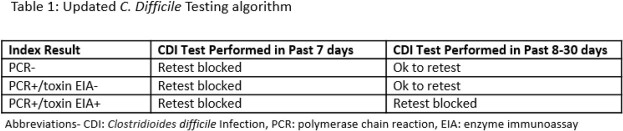

**Results:**

The HO-CDI standardized infection ratio (SIR) reduced significantly from 0.8 to 0.57 p< 0.001), and reportable HO-CDI cases reduced by 238 cases across the health system between May 2021 and March 2022. 6043 samples were tested, of which 282 (4.7%) were confirmed CDI cases (PCR+/toxin+) and 687 (11%) were non-CDI cases (PCR+/toxin-), of which 438 (67%) received CDI treatment. (Figure 2 and 3).

**Figure 3:**
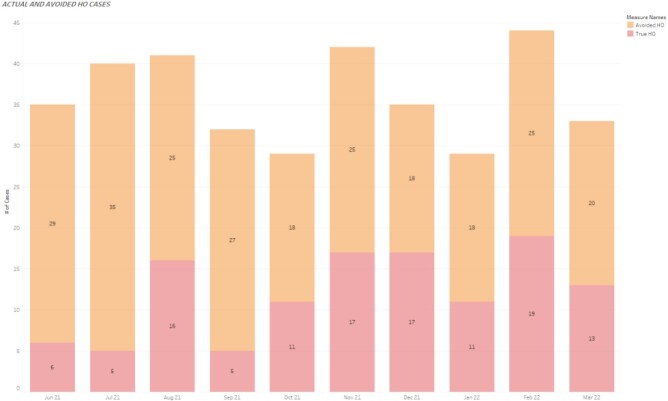
Actual versus avoided HO C. difficile cases.

**Conclusion:**

The two-step CDI diagnostic and treatment bundle significantly reduced the SIR of HO-CDI. Although treatment of colonized patients remained high, a large number of patients safely avoided CDI treatment. Testing and education bundles can help advance antimicrobial and diagnostic stewardship by improving detection, treatment, and tracking of CDI.

**Disclosures:**

**Asra Salim, MPH, CPH, FAPIC**, IRhythym Technologies Inc: Stocks/Bonds.

**Figure 2:**
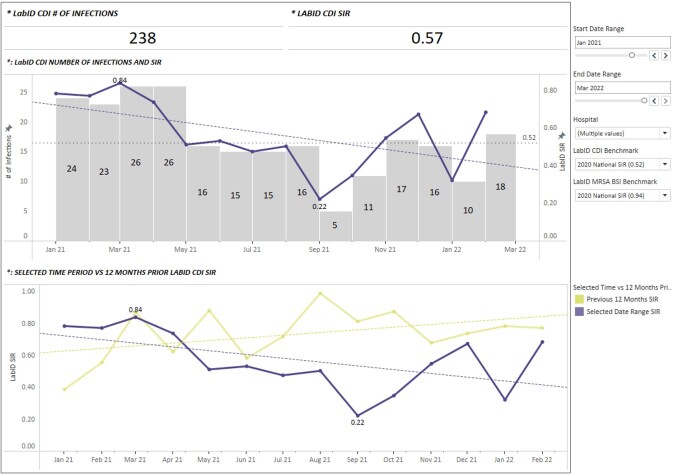
Rates of CDI SIR over the 12 month pre and post implementation period

